# Supplementary material for: Real-data-driven real-time reconfigurable microwave reflective surface
Source: Nat Commun. 2023 Nov 25;14:7736. doi: 10.1038/s41467-023-43473-y (PMC10676374; doi:10.1038/s41467-023-43473-y)
Supplement: Supplementary file 1 — Supplementary Information [file 41467_2023_43473_MOESM1_ESM.pdf]

Supplementary Materials for

Real-data-driven Real-time Reconfigurable Microwave Reflective Surface

Erda Wen[1]*, Xiaozhen Yang[1], Daniel F. Sievenpiper[1]

[1]Department of ECE, University of California San Diego; 9500 Gilman Drive #0407, La Jolla, CA, 92093, USA.

*Corresponding author. Email: ewen@ucsd.edu

The PDF file includes:

- Supplementary Note 1: Rigid-flex PCB Design
- Supplementary Note 2: Static Performance Measurement
- Supplementary Note 3: Curved Surface Configuration
- Supplementary Note 4: Pattern Data Collection & Data Processing
- Supplementary Note 5: Network Training and Performance
- Supplementary Note 6: 2-D Realization of Surface

Supplementary Note 1: Rigid-flex PCB Design

To demonstrate the challenge tunable flexible surface faces in microwave frequencies, Fig. S1. plotted the amplitude and phase response of the unit geometry that will is utilized in this design, on two substrates with different thickness.

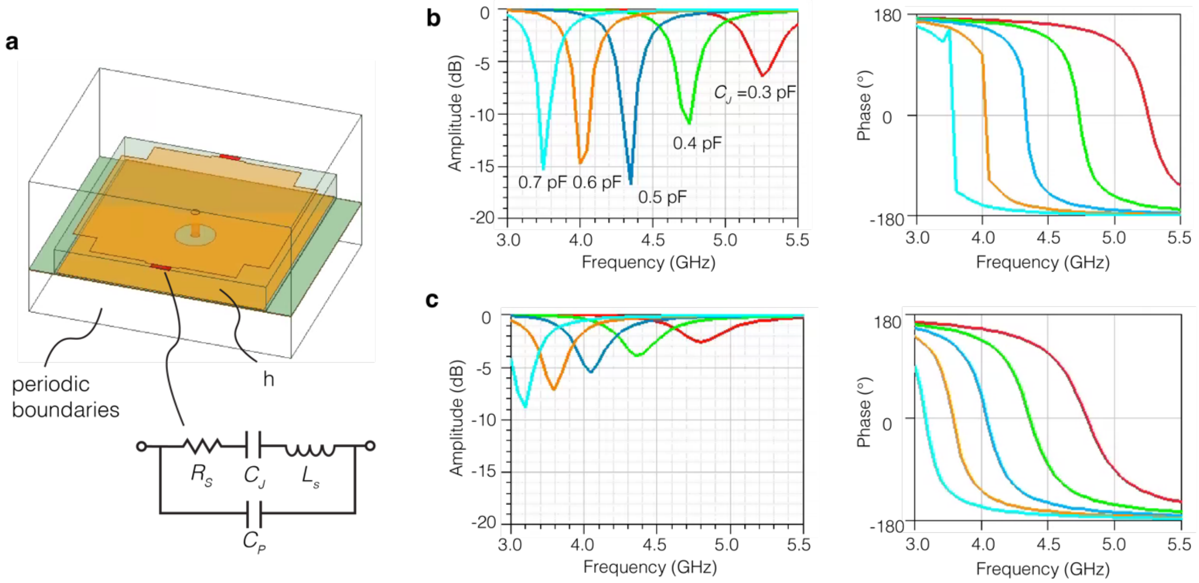

Fig. S1. RF loss on units. (a) Illustration of simulated model of a single unit under normal incidence (oblique incidence discussed in Note 3). The parasitic inductance, resistance and capacitance used in the simulation are: $L_s = 0.7$ nH, $R_s = 2.5\ \Omega$, $C_p = 0.1$ pf. (b) and (c) are reflection response of the unit with a thickness of $h =$ 30 mil and $h =$ 62 mil, respectively.

It can be observed that for thinner substrate:

1. efficiency decreases near resonance, and
2. quality factor of the resonance increases, causing a steeper slop in the reflection phase, increasing the sensitivity of the varactor.

Note that this limit is not only for this particular design but a rather general one (the Chu-Harrington limit is relevant if we consider the structure as an antenna). Given the relatively large parasitic resistance R_s of current varactor manufacturing technology, guaranteeing the electrical volume is critical to tunable metasurface designs.

To address this loss issue, we propose the prototype of a 4-layer rigid-flex design shown in Fig. S2. The rigid layer is made with 62 mil Rogers RT/Duroid 5880 material and the flexible layer is made with 2 mil polyimide substrate, bonded together with 6 mil prepreg. The unit periodicity is 13 mm along the column and 16.05 mm across the column with 3.05 mm separation in between columns. The top copper layer is protected with ENIG finish and the soldermask is only around

varactor footprints for soldering purposes. Ground plane copper layer and d.c. bias feeding network copper layer are protected with flexible coverlay.

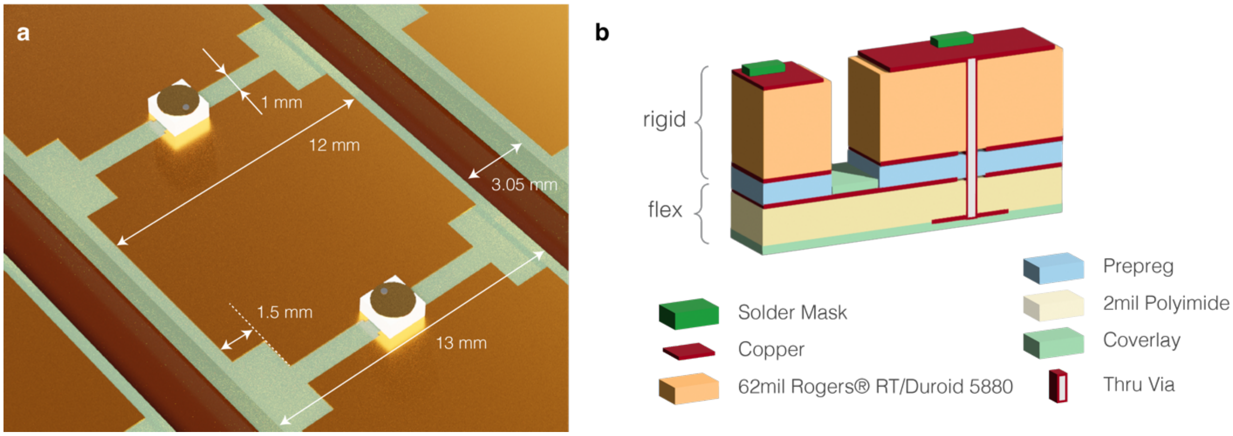

Fig. S2. Rigid-flex PCB design. (a) Dimension of the unit geometry. (b) Stack-up plot of the rigid-flex board. Dimensions are not proportional.

Supplementary Note 2: Static Performance Measurement

The static reflection spectrum of a flat metasurface is measured with a single horn RCDLPHA2G18B by RF-Lambda. S_{11} is recorded with an Agilent E5071C VNA for 3 cases:

1) bare horn, $S_{11,\mathrm{h}}$. This is the reflection from the horn itself;

2) an aluminum plate, as a perfect electrical conductor (PEC), of the same size, $S_{11,\mathrm{PEC}}$. This is the reflection from both the horn and a total reflection of a plane with a 180° phase shift, and

3) with metasurface in front of the horn, $S_{11,\mathrm{s}}$, all channels set from 0 V to 18 V. This is the reflection from both the horn and the surface.

The complex relative reflection on the surface is thus:

$$R = \frac{S_{11,\mathrm{s}} - S_{11,\mathrm{h}}}{S_{11,\mathrm{PEC}} - S_{11,\mathrm{h}}}$$

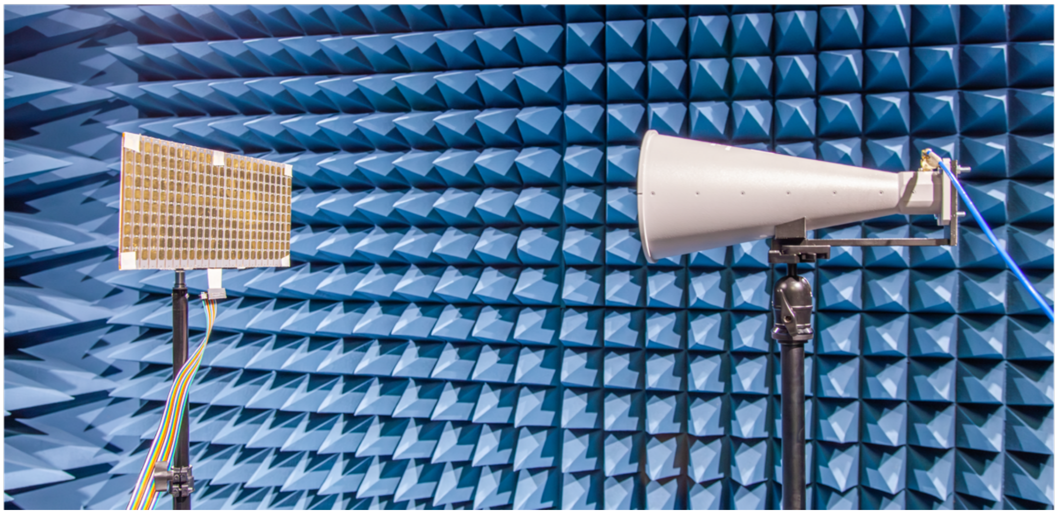

Fig. S3. Static reflection spectrum measurement. The distance between the horn aperture and the patch is 55 cm.

Supplementary Note 3: Curved Surface Configuration

For all four cases mentioned in the main text, the flexible surface is mounted on a 3-D printed backplane made of PLA plastic fastened with nylon screws. The curve surface for case B, C and D is a spline with 4 anchor points. The 2-D scattering object for case D is a 3-D PLA cylinder. The dimensions are given in Fig. S4.

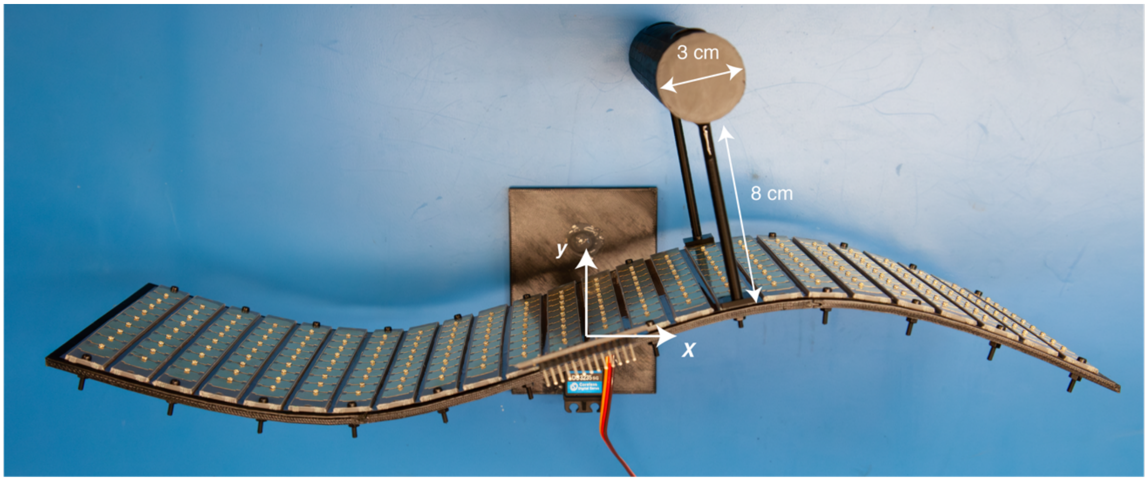

Fig. S4. Photo of the curved surface being measured. The anchor points for the spline curve are $[(-19.8,0),(-7.6,-3.0),(7.6,2.0),(19.8,0)]$, units in cm. The length of the cylinder on the z direction is 20 cm.

To demonstrate why simple analytical models like treating the surface as an antenna array would not work here, we calculate the reflection pattern of a PEC surface with:

$$D(\theta) = \frac{1}{\sqrt{n}}\sum_{i=1}^{n} e^{j(\varphi_{\mathrm{in},i}+\varphi_{\mathrm{out},i})},$$

where

$$\varphi_{\mathrm{in},i} = 2\pi r_i \cos(\theta_{inc} - \theta_i)/\lambda,$$

$$\varphi_{\mathrm{out},i} = 2\pi r_i \cos(\theta - \theta_i)/\lambda.$$

Fig. S5 shows the comparison of this calculation with the simulation results in ANSYS HFSS, on a flat surface and a curved surface used in the experiment. This estimation works well for flat

surface as expected but fail to match the curved surface, missing a bunch of features in the reflection pattern, due to the cross-talking of the elements.

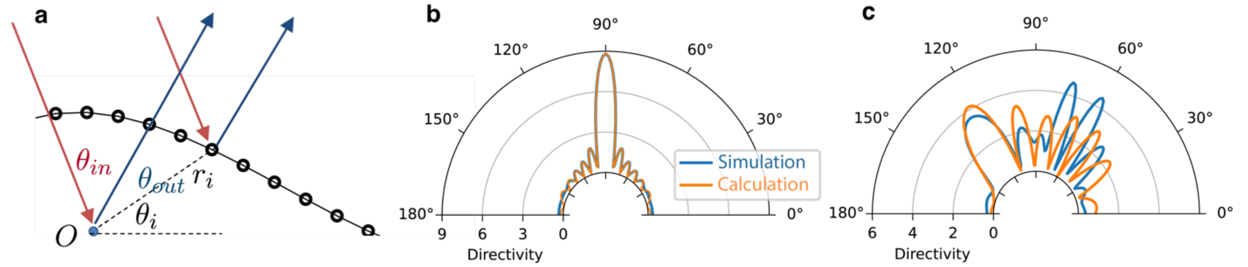

Fig. S5. Calculating a surface with simple array model. (a) The surface is being considered as multiple independent antenna array elements, and the array factor (AF) can be calculated by super-positioning the reflection of each element. Comparison between simulation and calculated pattern (normal incidence) of a (b) flat surface and (c) a curved surface same as in Fig. S4.

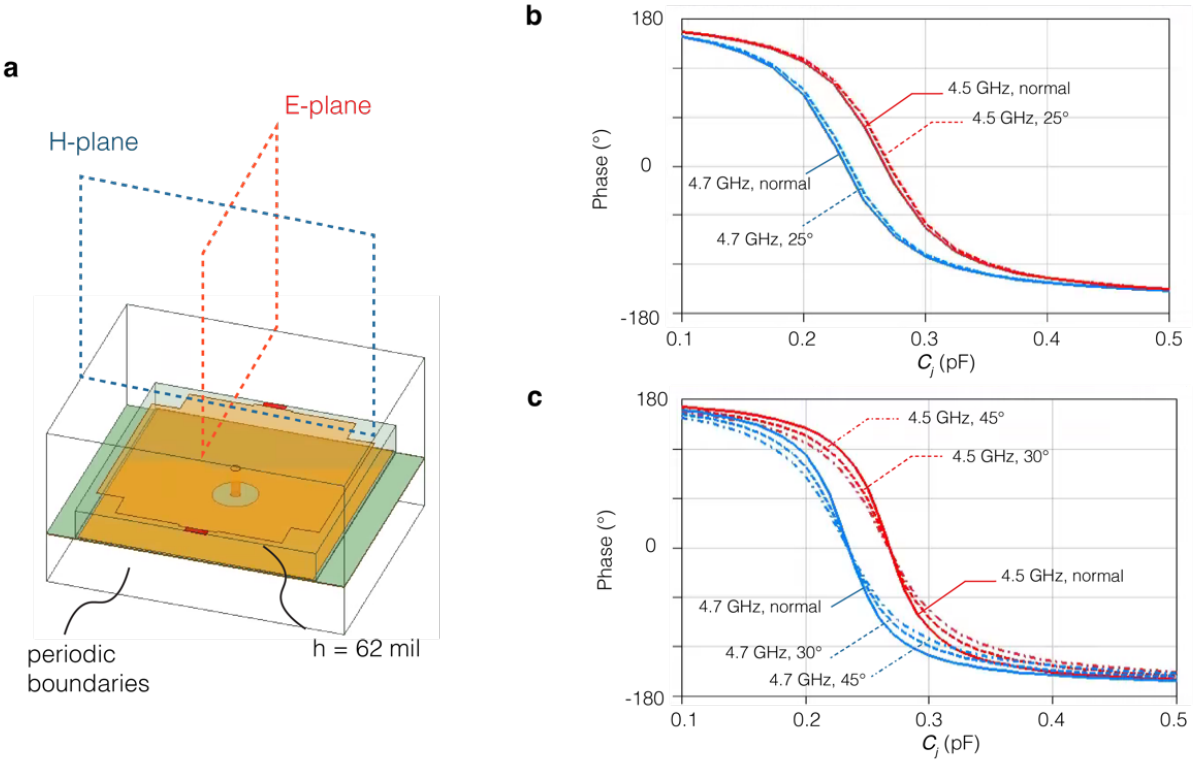

Fig. S6. Reflection phase response under different incident angle. Simulation results with Ansys HFSS. (a) E-plane and H-plane definition. (b) Comparison of reflection response under 0°, 25° on E-plane (c) Comparison of reflection response under 0°, 30°, 40° on H-plane.

Another effect that is worth discussing here is the dependency between reflection phase response and the incident angle, as shown in Fig. S6.

For E-plane, no noticeable dependency can be observed, which validate our setup that uses the specular reflection in this direction. For H-plane, slight dependency can be observed, which is another factor that potentially complicates analytical approaches, since in curved surface, each unit has different local incident angle and thus need its own phase-bias mapping.

Supplementary Note 4: Pattern Data Collection & Data Processing

The configuration of pattern measurement can be found in Fig. 3 in the main text. The distance between the Tx and the SUT is 75 cm and the distance between the SUT and the Rx is 190 cm. The specular angle in the elevation plane is 25°.

The test bench is controlled by a single controller PXIe-8135 by National Instruments (NI) running python 3.7. For the bias voltage supply, three 8-channel 16-bit DAQ cards NI PXI-6733 and a d.c. source Keithley 2410 are used. A servo motor ETS Lindgren 2005, attaching the SUT (with a tripod) and the Tx antenna (with a wooden arm), is employed to realize azimuth pattern scanning. Another small servo motor ZOSKAY DS3235SG is used to rotate the SUT to simulate incident angle changes. This motor is driven by an Adafruit FT232H breakout and a Adafruit 12-bit PMW driver. The interconnections between all equipment are shown in Fig. S7.

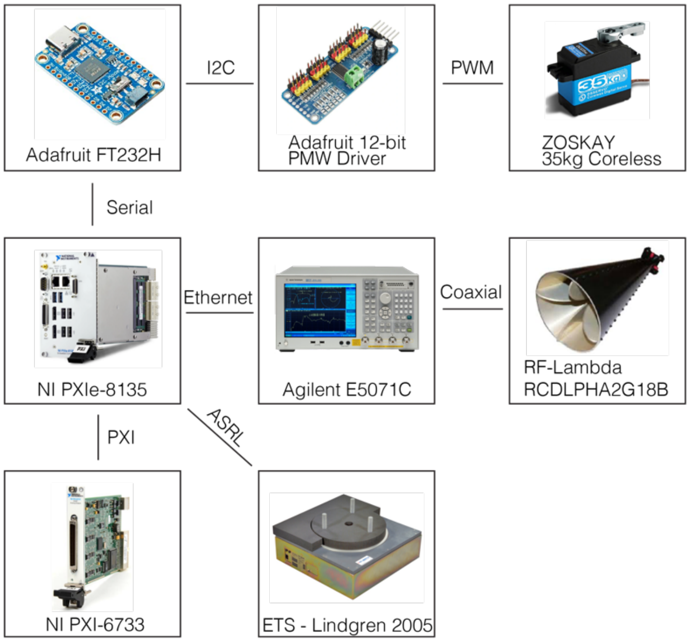

Fig. S7. Interconnections between the devices.

The transmission between the Tx and the Rx along the azimuth plane, from 3 GHz to 5.5 GHz, are measured for 3 cases:

1) blank case $S_{21,\mathrm{b}}$, where the surface is not mounted on the supporting structures. This includes the ambient noise, scattering from the supporting structures, and direct talk between Tx and Rx;
2) SUT case $S_{21,\mathrm{s}}$. This is the direct reflection along with all noises mentioned above.
3) aluminum board $S_{21,\mathrm{PEC}}$. This is for calculating the directivity of the board with the transmission intensity.

The loop of sampling, from outer to inner is:

1. θ scanning, by azimuth motor rotation.
2. θ_{inc} scanning, by incident motor rotation (for case C and case D).
3. Different bias voltages, by DAQs.
4. Frequency, done by VNA itself.

The wait time for step 1 and step 2 are set to 10 s and 4 s, respectively, to reduce the impact of mechanical vibration. Wait time between different sets bias is 20 ms.

To reduce the unwanted noise or environmental scatterings, all received signal is transferred to time domain with inverse Fourier transformation to apply a 5 ns time gate, then transferred back to frequency domain. Example of the time response in the normal direction is shown as in Fig. S8. From the plot, it can be estimated that the SNR with time gating but no blank-case calibration is at least 22 dB (closest peak difference between SUT and Blank case at around 16.7 ns), and with blank case calibration one could expect at least 30 dB SNR assuming the abient noise to be below -100 dB as the intensity away from the gated region indicates.

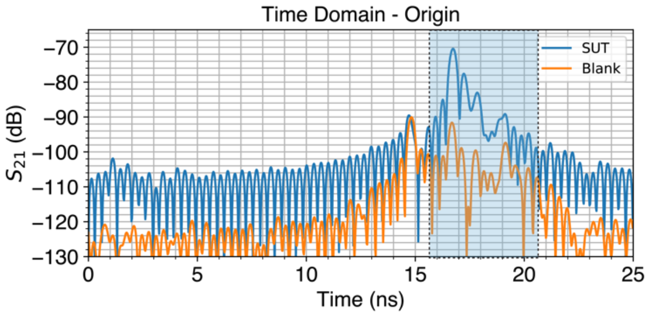

Fig. S8. Surface response in time domain. This example is from one sample in case A at $\theta = 90°$. The direct reflection from the SUT can be observed spanning from around 16 ns to 20 ns. The direct talk between the Tx and the Rx is likely the peak appears around 14.5 ns. Highlighted regions are the 5 ns window being applied.

The directivity is thus calculated by:

$$D(\theta) = \left| \frac{S_{21,\mathrm{s}}(\theta) - S_{21,\mathrm{b}}(\theta)}{S_{21,\mathrm{PEC}}(90°) - S_{21,\mathrm{b}}(90°)} \right| \times D_{\mathrm{PEC}}(90°),$$

where $D_{\mathrm{PEC}}(90°) = 8.85$ is the board-side directivity of a PEC aperture.

The RCS can be derived from the directivity with:

$$\sigma(\theta) = \left(\frac{D(\theta)}{D_{\mathrm{PEC}}(90°)} \right)^2 \times A_{\mathrm{PEC}},$$

where A_{PEC} is the physical aperture of the PEC plate.

Supplementary Note 5: Network Training and Performance

Fig. S9 illustrates the detailed architecture on the sequential tandem neural network used for this work.

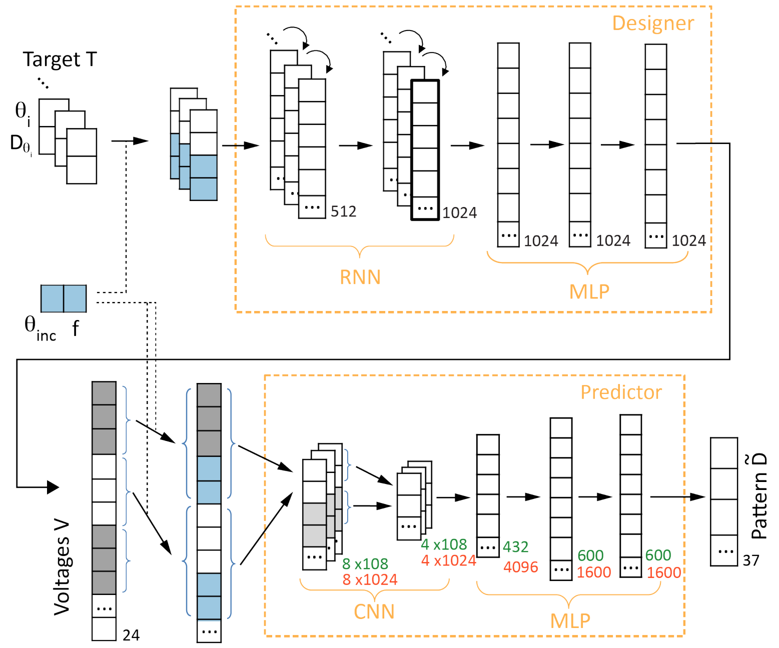

Fig. S9. Proposed sequential tandem network architecture. Dimensions of tensors in the predictor for case A, B and case C, D are in green and red, respectively.

All data, including bias voltages, directivity intensity, incident angle, frequency and target directions are all normalized to within [0,1] in the training process. To deal with overfit, L2 regularization is used. The parameter is optimized with Adam optimizer. The batch size for training is set to 50. Table S1. Listed the detailed hyperparameter used for four cases.

Table S1. Predictor training hyperparameters.

Case	A	B	C	D
L2 weight	2e-7	5e-7	5e-8	5e-8
Learning rate	1e-3	1e-3	3e-4	3e-4
Epochs	500	500	50	50

The loss function vs. epochs is plotted in Fig. S10. The parameters of the epoch with the lowest validation loss are being used.

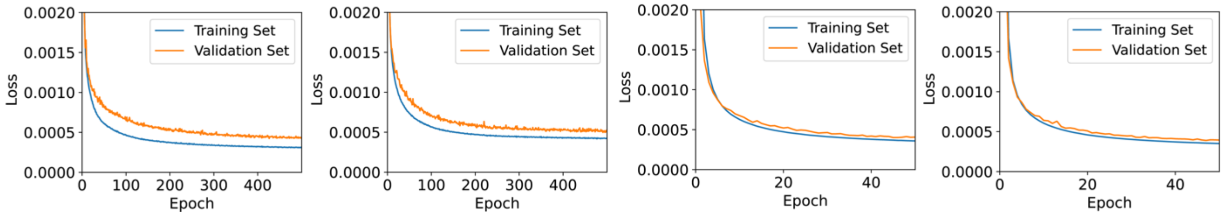

Fig. S10. Training process of the predictor. From left to right are losses for case A to case D, respectively.

The overall performance of the trained predictor is shown in Fig. S11.

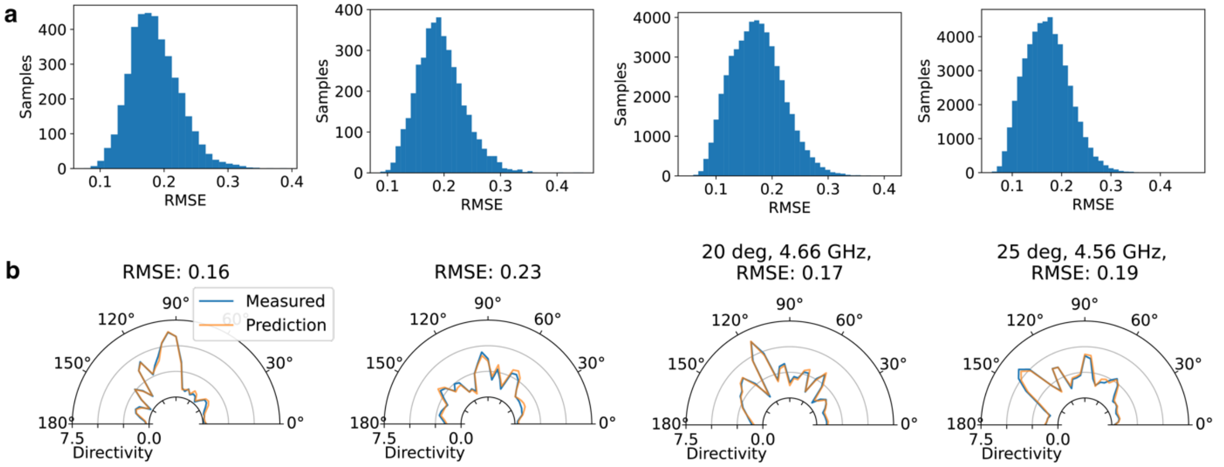

Fig. S11. Performance for the predictor for four cases. (a) RMSE distribution from case A to case D, in the test set. (b) Visual examples with typical error level.

The parameter from the predictor is then inherited and fixed during the training of the designer. In designer, no regularization is used since it is always preferred to generate more data to address overfitting and improve generality. Considering the energy distributing effect for multiple targets, the range of directivity on goals with different target number is listed in Table S2.

Table S2. Directivity range for different number of targets.

# of targets	1	2	3	4	5
Max directivity	8.85	6.25	5.10	4.43	3.96

The batch size for training is set to 50. The hyperparameter for training the network is listed in Table S3.

Table S3. Designer training hyperparameters.

Case	A	B	C	D
Learning rate	4e-5	6e-5	5e-5	6e-5
Epochs	150	150	120	200

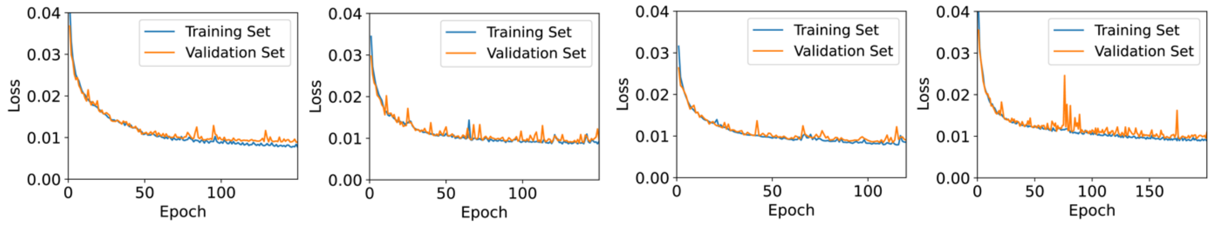

Fig. S12. Training progress of the designer. From left to right are losses for case A to case D, respectively.

Fig. S13-S16 give RMSE distribution and visual examples for the designer performance on random samples for case A to case D, respectively, while Fig. S17 give some examples on commonly used practical tasks like beam and null steering.

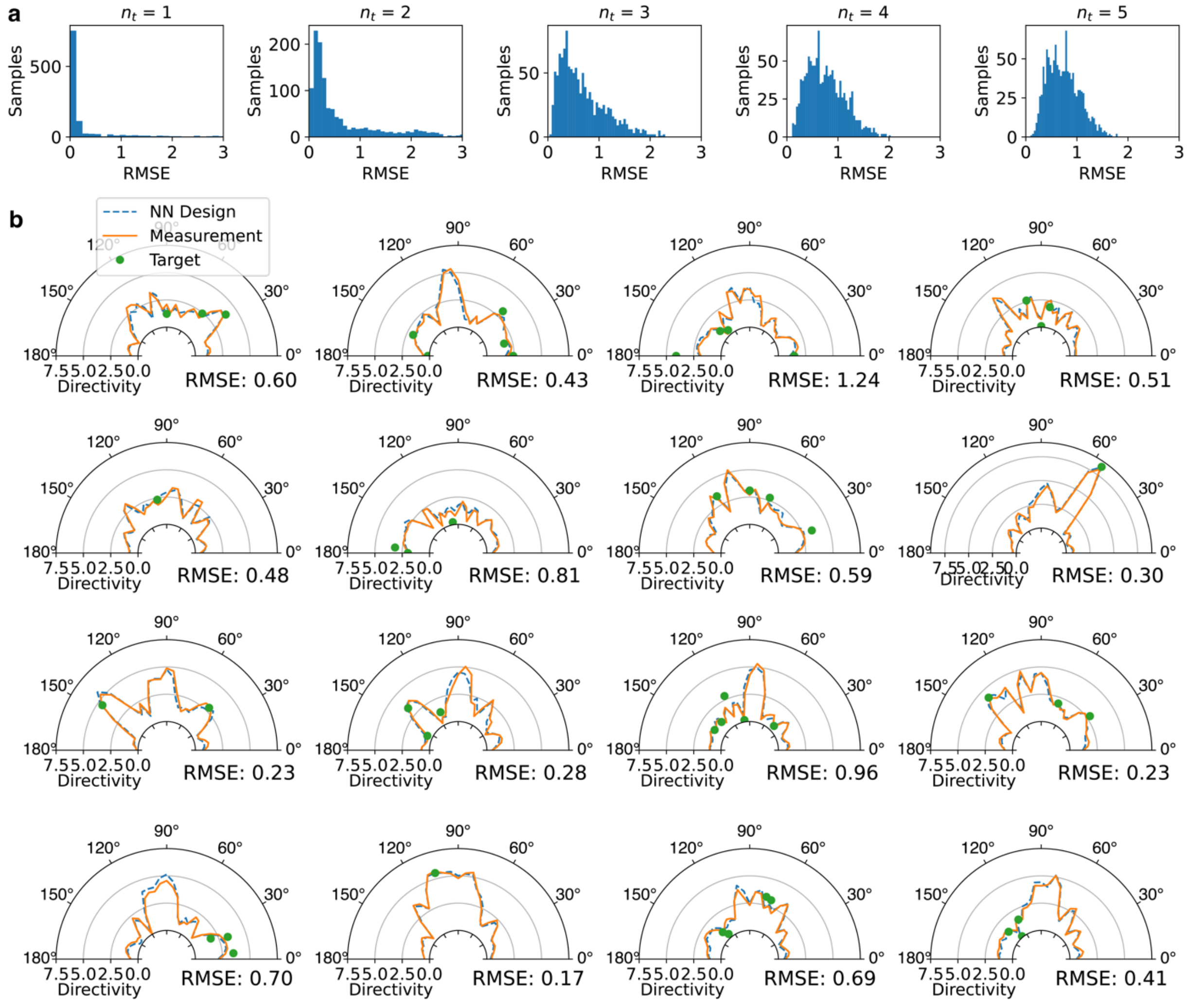

Fig. S13. Performance of the designer for case A. (a) RMSE distribution on sequence with different target number n_t. (b) Random-selected visual examples, sampling from 6,000 test samples with random seed = 1. Normal incidence with frequency 4.7 GHz.

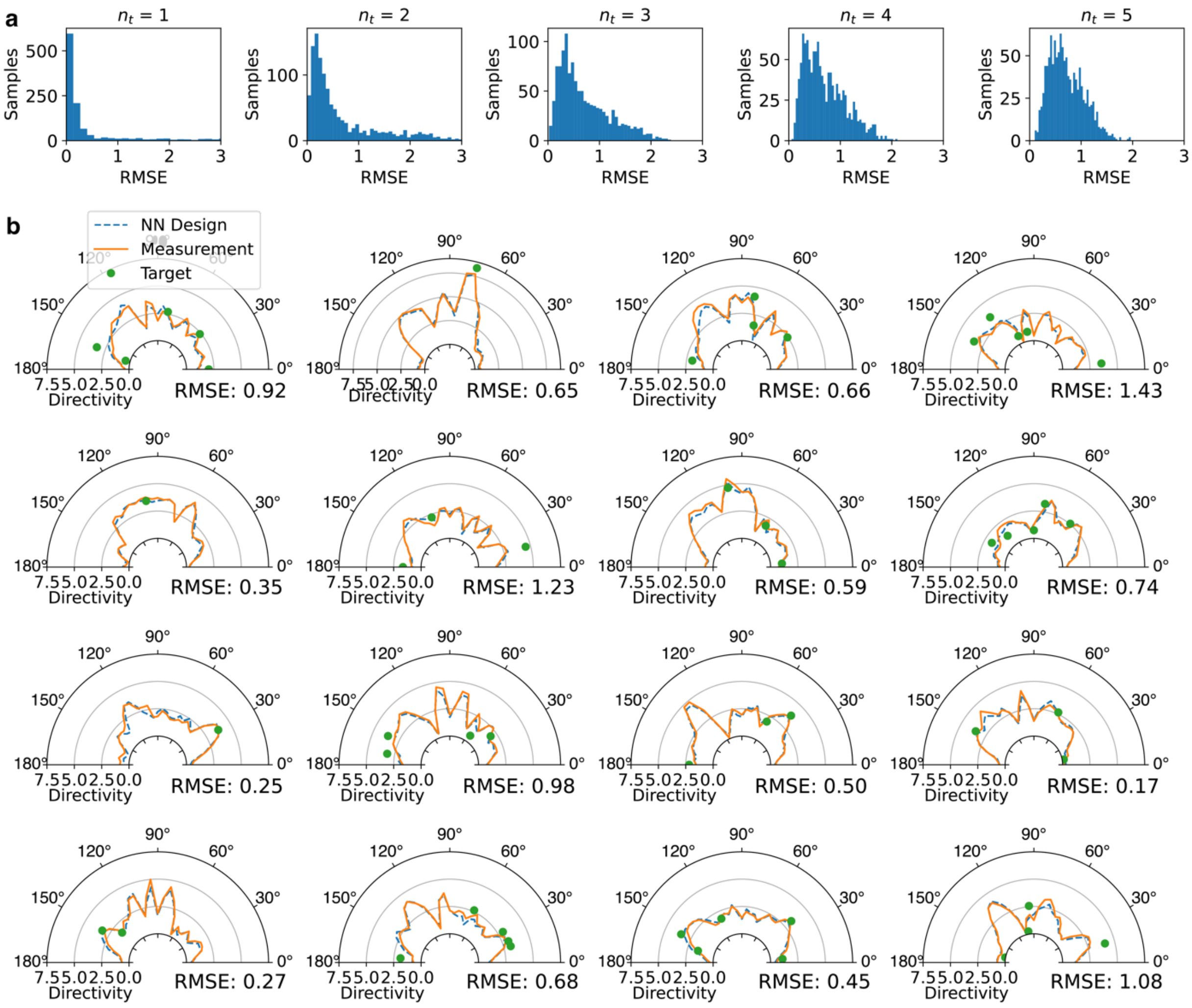

Fig. S14. Performance of the designer for case B. (a) RMSE distribution on sequence with different target number n_t. (b) Random-selected visual examples, sampling from 6,000 test samples with random seed = 2. Normal incidence with frequency 4.7 GHz.

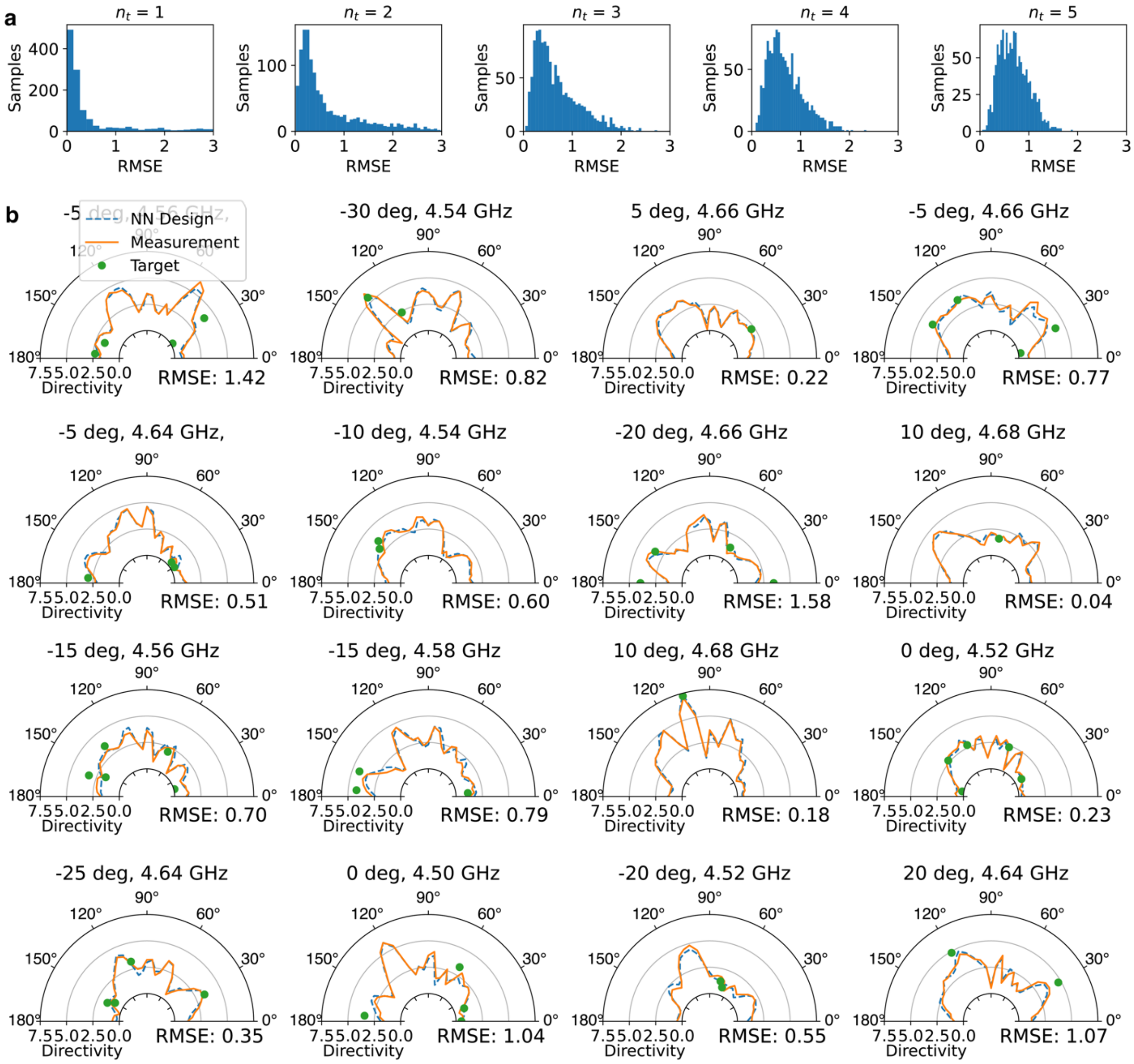

Fig. S15. Performance of the designer for case C. (a) RMSE distribution on sequence with different target number n_t. (b) Random-selected visual examples, sampling from 6,500 test samples with random seed = 3. The incident angle and operating frequency are noted on top of each pattern.

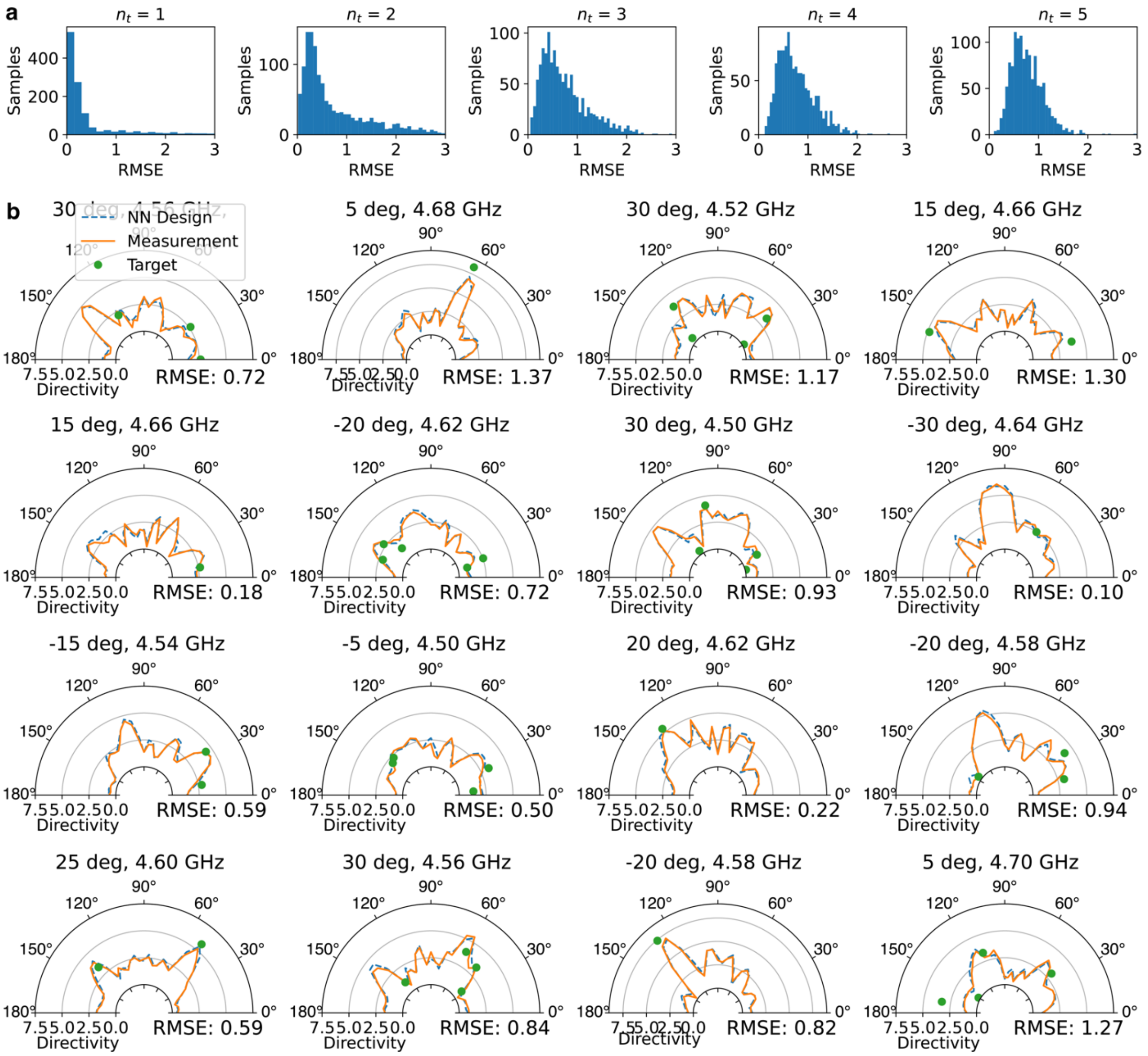

Fig. S16. Performance of the designer for case D. (a) RMSE distribution on sequence with different target number n_t. (b) Random-selected visual examples, sampling from 6,500 test samples with random seed = 4. The incident angle and operating frequency are noted on top of each pattern.

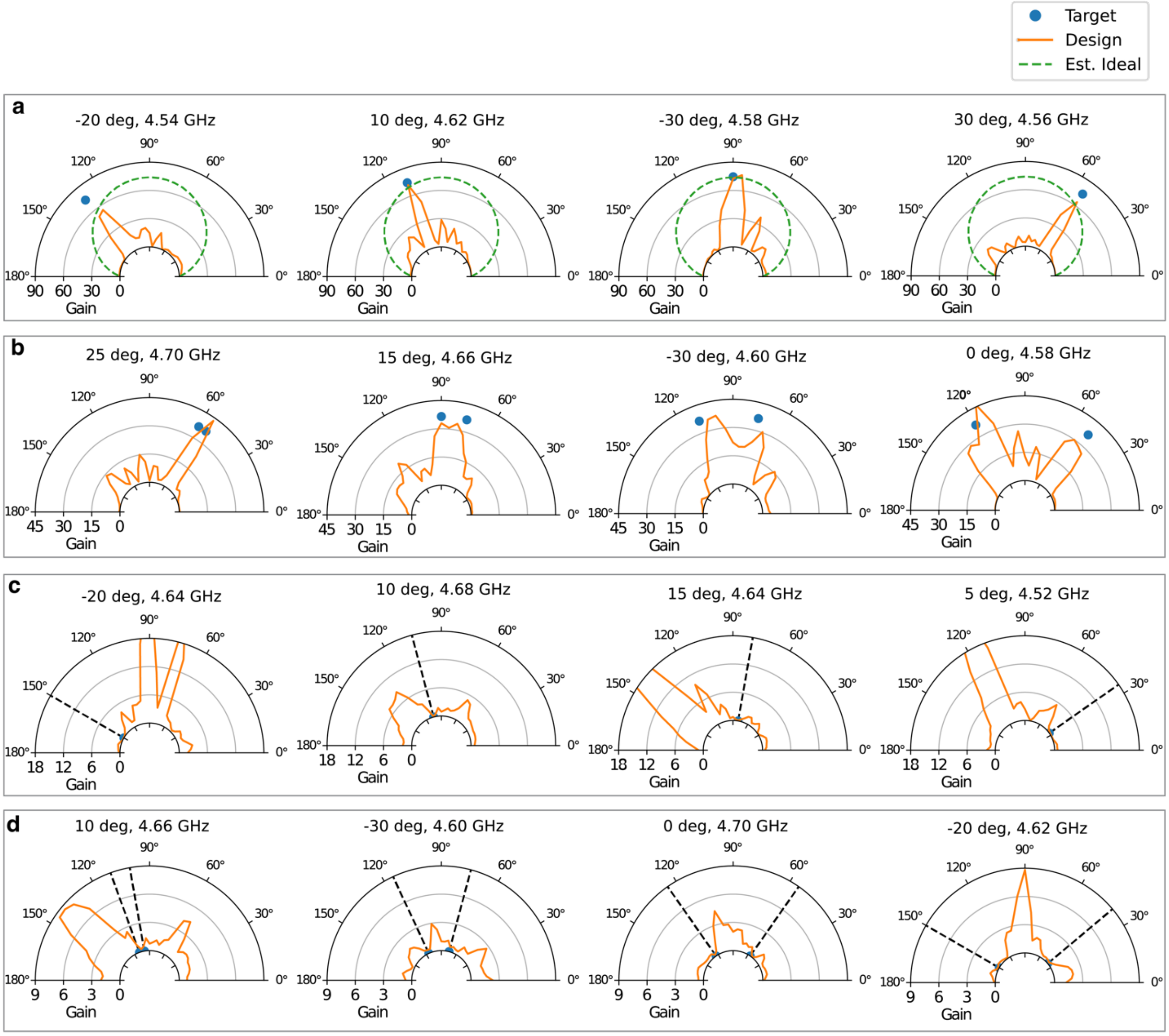

Fig. S17. Power gain plot of beam/null steering, under case C. The incident angle and operating frequency are noted on top of each pattern. (a) One beam sweeping to different directions. Dashed green curve is a $\sin\theta$ envelope that estimates the maximum feasible gain towards each direction, due to decreeing projected radiation aperture from broadside direction. (b) Two beams with various separation. (c) One null sweeping to different direction. Dashed black lines are the target null directions. (d) Two nulls with various separation.

On the scalability of the network, it is reasonable to estimate that for the prediction network, since CNN is being used, should have a scalability of $O(\log(n))$, and for the designer, a linear $O(n)$ relationship, since it uses fully connected layer. However, proving evidence for this claim will need very comprehensive analyses and very carefully designed experiment, which is out of the scope of this work, but should make very good future study.

Supplementary Note 6: 2-D Realization of Surface

In this study we use a patch-antenna-like resonance structure as our unit design, which may not be preferable for 2-D cases since the varactor is sitting on the edge between neighboring ceils on E-plane. Nevertheless, the same stack-up structure can be used for other unit geometry design, including designs with lumped element positioning more suitable for 2-D realizations.

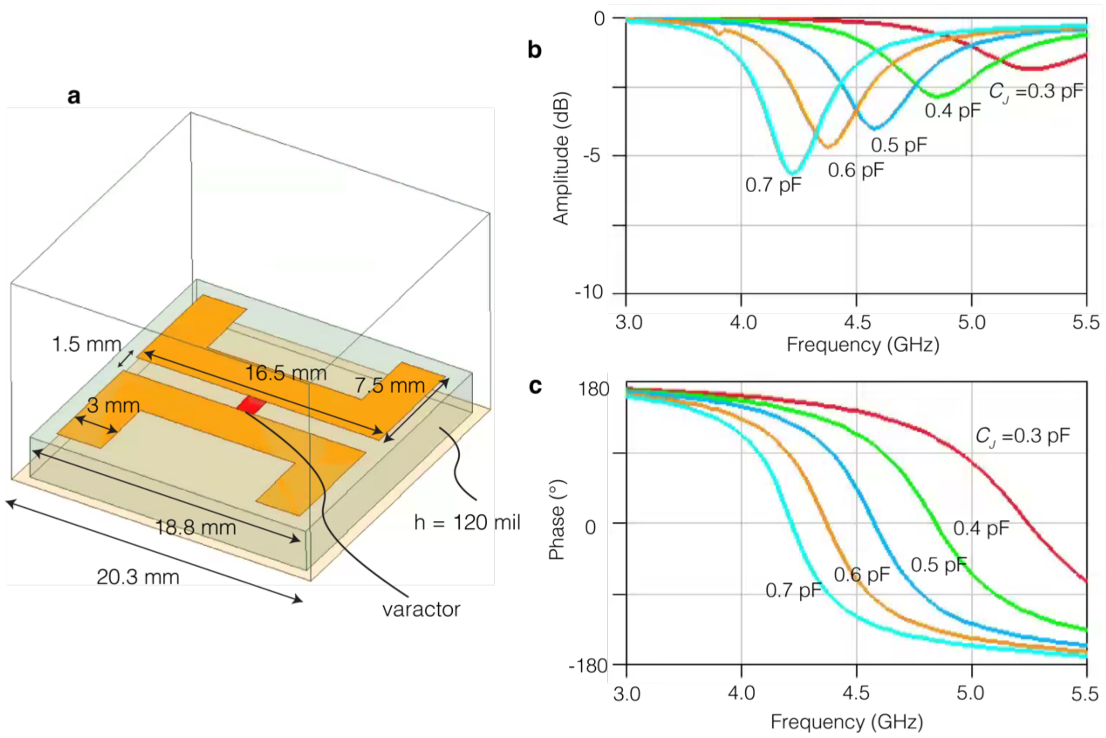

Fig. S18. A modification of units in [30]. (a) Sketch of the unit, with Rogers RT/Duroid 5880 as the substrate. (b) and (c) are amplitude and phase response of the unit.

In Fig. S18, we take unit design in [30] as an example, by tuning the dimension, the unit can work within a frequency range around what in this study, with gaps of rigid board around all four directions. Notably, it requires larger volume than our design since it does not take full advantage of the unit aperture. Yet, the size is still small enough to prevent grating lobe its operating frequencies.
